# A Case of Plastic Surgery

**Published:** 1883-11

**Authors:** A. G. Fredericks


### A Case of Plastic Surgery.

BY A. G. FREDERICKS, M.D.

As an introductory to the relation of a case of plastic surgery, which came under my care, and upon which I have operated, I think it pertinent to give a brief sketch of the various methods which have been resorted to, to relieve the different deformities which have been occasioned by accident or disease.

The performance of any operation for the repair of a loss of structure, whether resulting from a disease or accident, is called plastic surgery.

Portions of the body that have been partly detached may retain a sufficient amount of vitality to become adherent even should it be but a small and narrow strip of tissue by which they are attached to the part from which they have been separated. Many no doubt have observed this in enjuries of the face and fingers, portions of which have been nearly or intirely severed after being replaced have united again. Also there have been a great number of cases on record wherein certain parts have been completely separated upon being replaced became adherent.

Prof. Hoffacher, whose correctness is attested by Chelens and Valpeau, relates the most remarkable instances of this kind, being officially appointed to attend as surgeon to the many duels which occured at Heidelberg amongst the students. These duels were very prevalent at that time and the weapons used were broad-swords; so, consequently, he had an excellent opportunity of seeing a considerable number of incised wounds; reports not less than sixteen cases in which portions of the nose, lip, or chin had been completely sliced off, and on being replaced became again adherent. Amongst the number was a case where the nose was cut off and lost, and it was sometime before it was found; when recovered it was washed and stitched on and became attached. Another time a dog happened to be in the room who snapped up the poor fellow's nose, but the portion was recovered whole from the dog's mouth, on being washed and applied it, united.

Success to attend cases of plastic surgery, the parts between which union is to take place, whether separated completely or nearly so, to the rest of the body, should be of a homogeneous character soft and where there are no large blood vessels, nerves, tendons or bones, but in tissues such as is met with in the face. Of course the same rule applies in plastic operations, and likewise most success attends where there are similar conditions of tissue and which are conducted upon the same principle as an attempt at union in a partially severed structure.

A fleshy knob which brings woe to the possessor who carries it on his shoulders and is a horror to every one else is converted by plastic surgery into a human physigonomy and gives back to the discarded and avoided person both life and society; the eye, which deprived of its natural covering becomes dry and inflamed, would withdraw itself, with convulsive efforts, but in vain from the destructive influence of light and air, seeking the repose which avoids it, is covered again with protective eyelids and regains life and sleep. A mouth puckered up, grown together like an eyelet hole for which no food is suitable save soups and thin broths and whose expulsive articulations resemble the cry of a wild beast, again gives forth human sounds, reopens and allows the patient both to eat and speak.

And let me add, have I yet cause to say that plastic surgery has the power to heal both urinary and fecal fistula, and thus to remove the greatest physical suffering with which man is afflicted in this world. Thus graphically has Dr. Fritze described the benefits which flow from plastic surgery.

Plastic surgery is most serviceable to relieve deformities of the nose and lips, and certainly there is hardly any deformity more abhorent than a deficiency in either of the foregoing, and which test the skill of the surgeon.

Rhinoplasty can be accomplished by two methods, the one by taking the flap from the fore-arm or from another person, the other by taking the flap from the surrounding tissue, as, for instance, the forehead.

You have also operations for the motion of the eyelids called blephoraplasty, to relieve entropium, ectropium, and closure of lachrymal sac; also staphyloraphy to correct cleft palate, not an advisable operation especially upon the hard plate. An obturator is far more preferable and can remedy the defect very satisfactorily and with a great deal less trouble.

None of us are very likely to be called upon to operate for the relief of a ruptured perinæum or a urinary or fecal fistula, so I will not burthen you with a discription of them or the different methods of operating.

Cheiloplasty, the operation to relieve a defective lip, is as various and multiple as the ingenuity of the operator might suggest, as there are hardly any two cases exactly alike.

The case to which I will now refer is one of cheiloplasty. My patient, a woman aged about thirty-five years, who had lost by disease (tertiary syphilis) the palatal bones, vomer, and a portion of the superior maxillary with its alveolar process from the second bicuspid on the left side to the first molar on the right, the soft parts having sloughed away left a triangular opening about two inches at its base with its apex toward the nose, which was sunken in and with still considerable ulceration in her pharynx. Such was her condition when I first saw her. Her previous history was quite conflicting. She attributes all her suffering to a blow she had received some three years previous to her marriage. From what I could learn from other sources, she bore a good character as being a sober and hard-working girl. Three years after her marriage, which occured some twelve years ago, her trouble began to make its appearance by a slight swelling in the nose, continuing its ravages during a period of six years and resulting in the loss above described , since which date (with exception of the ulceration in the throat) she has had no further exhibition of the disease. I immediately put her under syphilitic treatment and at the end of a few weeks all ulceration entirely disappeared. As the case presented itself it was apparent to my mind that nothing could be done for her but to make an artificial lip out of the surrounding soft tissue, for the use of any hard material instead of accomplishing this end would only result in making her disfigurement more conspicuous. To make an obturator with teeth would only "add insult to injury." I informed her that her only chance for a betterment was to submit to a cheiloplastic operation, adding that if she desired it to be done she must assume all consequences, as I could guarantee only my best efforts to benefit her.

After a month's delay she consented. Upon the Sunday following, with the kind assistance of Drs. Loeber, Souchon, and others, I operated. I proceeded as follows: Placing the patient under chloroform I began by making a triangular flap on the left side, paring the edges on the right and cutting a triangular piece of tissue off of its end, dissecting the tissue underneath up as high as the orbital ridge and backward to the zygomatic process, then downwards on the other side—the parts throughout being bound down by a dense cicatricial tissue—a resultant of the previous inflammation. In drawing the parts together I used four silver suture pins with figure of 8 suture and at the edge three silk thread sutures. Three days after I was able to remove all the sutures, and within three weeks the desired result was achieved.

[*A photograph accompanied this description, which shows a very successful operation.*—ED.]

